# Anthelmintic effect of *Indigofera tinctoria* L on *Haemonchus contortus* obtained from sheep in Indonesia

**DOI:** 10.14202/vetworld.2021.1272-1278

**Published:** 2021-05-22

**Authors:** Iskandar Muda, Joko Prastowo, Wisnu Nurcahyo, Sarmin Sarmin

**Affiliations:** 1 Doctoral Study Program of Veterinary Science, Faculty of Veterinary Medicine, Universitas Gadjah Mada, Bulaksumur Sleman, Yogyakarta 55281, Indonesia; 2 Animal Husbandry Training Center – Batu, Jl. Songgoriti 24 Batu, East Java, 65312, Indonesia; 3 Department of Parasitology, Faculty of Veterinary Medicine, Universitas Gadjah Mada, Bulaksumur Sleman, Yogyakarta 55281, Indonesia; 4 Department of Physiology, Faculty of Veterinary Medicine, Universitas Gadjah Mada, Bulaksumur Sleman, Yogyakarta 55281, Indonesia

**Keywords:** anthelmintic, *Haemonchus contortus*, *Indigofera tinctoria*, scanning electron microscopy, sheep

## Abstract

**Background and Aim:**

*Hemonchosis* has resulted in huge economic losses for sheep farmers worldwide. Secondary metabolite compounds from *Indigofera tinctoria* L. can be used as anthelmintics. This study aimed to evaluate the *in vitro* and *in vivo* effects of *I. tinctoria* L. aqueous extract (IAE) as an anthelmintic against adult *Haemonchus contortus* isolated from sheep.

**Materials and Methods:**

Ten active adult worms were placed in each Petri dish containing 25 mL of IAE, each having a different concentration of IAE (at concentrations 100, 120, 140, 160, 180, 200, and 220 mg/mL). Each experiment was repeated. The positive control used 1% albendazole, and the negative control used 0.62% saline water. The number of immobile worms and the time of mortality were recorded after 2, 4, 6, and 8 h. The dead worms were subsequently tested using scanning electron microscopy (SEM) and sodium dodecyl sulfate-polyacrylamide gel electrophoresis. In the *in vivo* study, 15 sheep with an average fecal egg count (FEC) of 1000 eggs per gram were treated with IAE for 9 days: Group A (negative control, saline water 0.62%), Group B (21 mg/mL), Group C (41 mg/mL), Group D (62 mg/mL), and Group E (positive control, albendazole 100 mg/mL). Measurements of the body weight, FEC reduction test, and hematology testing were performed on days 0, 7, and 14. SEM was performed using worms found from the abomasum of slaughtered sheep.

**Results:**

The leaves of *I. tinctoria* L. contained a number of secondary metabolites, including total tannins, saponins, flavonoids, and alkaloids. The most effective concentration that killed the adult *H. contortus* worms was 220 mg/mL (93.33% mortality) after 8 h of treatment. The electrophoresis results showed that the protein band at a dose of 22% was less than that of the control. The highest FECR value of the treatment group on the 14^th^ day after treatment was at a dose of 62 mg/mL. The highest weight gain as well as the highest increased hemoglobin (Hb), packed cell volume (PCV), and total erythrocyte count (TEC) values on the 14^th^ day after treatment were at a dose of 41 mg/mL. The SEM results showed that IAE treatment caused the worms’ anterior parts to become wrinkled with thick creases and cuticle abrasion (*in vitro*) and the anterior part to shrink along with the presence of aggregates in the worm cuticle (*in vivo*).

**Conclusion:**

The aqueous extract of *I. tinctoria* contains tannins, saponins, flavonoids, and alkaloids and has an anthelmintic effect with decreased FEC, increased weight gain, Hb, PCV, and TEC, causing damage to the worms’ body and reducing the protein profile of adult *H. contortus* worms.

## Introduction

*Haemonchus contortus* is a blood-sucking gastrointestinal worm from the nematode class (Familia: Trichostrongylidae). In the sheep husbandry business, *hemonchosis* has caused huge economic losses. *H. contortus* infestation disrupts the health of farmed sheep in Indonesia. In sheep, *hemonchosis* can cause mortality, decreased production, stunted growth, and low body weight [1].

The prevalence of *hemonchosis* in sheep is still quite high. Baihaqi *et al*. [2] stated that the prevalence of *Haemonchus* sp. in thin-tailed sheep in Wonosobo, Indonesia, was 55.41%. Abdo *et al*. [3] stated that the *hemonchosis* infection rate in sheep slaughtered at a slaughterhouse in Bishoftu, Ethiopia was 69.6%, with a prevalence in young and adult cattle of 66.9% and 59%, respectively. Bibi *et al*. [4] reported that the prevalence of *hemonchosis* in sheep in Pakistan was 55%. Secondary metabolite compounds from plant extracts can be used as anthelmintics. *Indigofera tinctoria* L. is very commonly grown and can be easily found in Indonesia. Kusumawati *et al*. [5] stated that *I. tinctoria* contains tannins. Renukadevi and Sultana [6] added that the leaf extract of *I. tinctoria* contains flavonoids, saponins, sterols, terpenoids, phenolic acids, quinones, and tannins. Some plants that are the sources of tannins have an anthelmintic effect on nematodes [7]. Maestrini *et al*. [8] added that saponins also have an anthelmintic effect. Tannin and flavonoids have effective anthelmintic effects against worm eggs and larvae [9].

This study aimed to evaluate the *in vitro* and *in vivo* effects of *I. tinctoria* L. aqueous extract as an anthelmintic against adult *H. contortus* isolated from sheep.

## Materials and Methods

### Ethical approval

This study was approved by the Institutional Ethical Committee, Faculty of Veterinary Medicine, Universitas Gadjah Mada, Yogyakarta, Indonesia (No. 0016/EC-FKH/Int/2019).

### Study period and location

The study was conducted from April 2019 to October 2020. All samples were processed in the Parasitology Laboratory of Veterinary Faculty of Gadjah Mada University, except for scanning electron microscopy (SEM) samples that were processed in the Laboratory of Indonesian Institute of Sciences.

### Plant collection and extraction

The leaves of *I. tinctoria* L. were obtained from Maguwoharjo Village, Depok District, Sleman Regency, Yogyakarta (7.46’43″ “S, 110.23’21”″ E, and 140 MASL). The plant specimens were identified at the Plant Taxonomy Laboratory of the Biology Faculty, Universitas Gadjah Mada, Yogyakarta, Indonesia. A number of *I. tinctoria* L. leaves were cleaned, dried in a drying room, and stored until they were used for the extraction process. Each of the dry *I. tinctoria* leaves (10, 12, 14, 16, 18, 20, and 22 g) was placed in 100 mL of distilled water and then incubated at 90°C for 15 min. The solution was filtered using filter paper, and the filtrate (100, 120, 140, 160, 180, 200, and 220 mg/mL) was stored in the refrigerator until SEM and sodium dodecyl sulfate-polyacrylamide gel electrophoresis (SDS-PAGE) were conducted [10].

### Determinination of qualitative plant phytochemical

The content of secondary metabolites in *I. tinctoria* leaves was determined using the spectrophotometric method. The total equivalent tannic acid was determined as described previously [11,12]. Total flavonoids were determined using the spectrophotometric method [13]. The results of the total equivalent tannic acid, saponins, flavonoids, and alkaloid equivalent quinine were expressed as %w/w (weight of tannins or flavonoids or saponin or alkaloid per weight of sample × 100).

### *In vitro* study

The *H. contortus* worms were obtained directly from the abomasum of naturally infected sheep slaughtered at a slaughterhouse in Sleman, Yogyakarta, Indonesia. This abomasum was taken to the Parasitology Laboratory of the Faculty of Veterinary Medicine, Universitas Gadjah Mada, Yogyakarta. The worm collection was done by opening the abomasum and cutting the curvature. Subsequently, the abomasum contents were poured out carefully, and the visible worms were collected and put into a container containing 0.62% physiological NaCl solution [10].

The *I. tinctoria* L. aqueous extract (IAE) was prepared 1 day before the worm mortality test according to the treatment dose. Ten active adult worms were placed in a Petri dish containing 25 mL of IAE. This experiment with each treatment group (negative control, 100, 120, 140, 160, 180, 200, and 220 mg/mL IAE, and positive control) was repeated. The positive control was administered albendazole 1%, and the negative control was administered 0.62% physiological NaCl. Observations were made after 2, 4, 6, and 8 h. The time of mortality and the number of immobile worms were recorded. Worm mortality was characterized by worms that did not move when the Petri dish was shaken or touched with a needle. The dead worms were put in an absolute ethanol solution and stored at 4°C until they were used for SEM and SDS-PAGE [10].

### *In vivo* study

Fifteen sheep with an average fecal egg count (FEC) of 1000 egg per gram (EPG) that was infected with L3 *H. contortus* were treated with IAE for 9 days: Negative control (saline water 0.62%), Group B (21 mg/mL), Group C (41 mg/mL), Group D (62 mg/mL), and positive control (albendazole 100 mg/mL). The variables analyzed included body weight, EPG, hematology results, and SEM. Observations, data collection, and testing were carried out on days 0, 7, and 14 [14]. SEM was performed using worms found in the abomasum of slaughtered sheep.

### SEM

Adult worms that had died from the anthelmintic test (*in vitro*) and adult worms found in the abomasum (*in vivo*) were used in this test. The worm samples were fixed in absolute ethanol. Subsequently, the following were carried out: Solid specimen preparation by cleaning in a cacodylate buffer for 2 h, prefixation in 2.5% glutaraldehyde for 48 h, fixation in 2% tannic acid with four washes in cacodylate buffer and distilled water, dehydrated in graded alcohol, and dried in tertiary butanol. Dry samples were coated with copper for 15 min and observed with SEM (JEOL JSM-5319LV, JEOL USA, Inc.) [15].

### SDS-PAGE

This test used adult worms that died from the anthelmintic test. The specimens were washed several times using phosphate-buffered saline (PBS) to remove debris and host-derived materials. The specimens were crushed in 100 uL of PBS. The dissolved protein was isolated through 1200× centrifugation for 5 min and used for electrophoresis. Electrophoresis was carried out based on the method used by Sambodo *et al*. [10]. Dissolved protein samples and markers (Thermo Scientific, Lithuania) were boiled for 2 min in a sample buffer with a volume ratio of 4:1 containing 50% glycerol, 10% SDS, 2.5% DTT, and 0.05% (w/v) bromophenol blue liquid as a marker dye. Electrophoresis was carried out at 100 V in a vertical slab gel system. After electrophoresis, the gel was stained with 0.2% (w/v) Coomassie blue.

### Fecal egg-count reduction test (FECRT)

Fecal sample collection was performed directly from the rectum. The EPG calculation was carried out using the McMaster egg counting technique (flotation procedures). The percentage decrease in the number of worm EPG of feces or FECR was calculated according to the following:


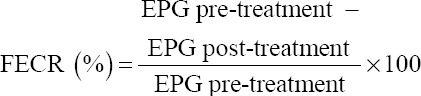


### Hematology

Blood was collected from all sheep through the jugular vein (2 mL/sheep). The parameters analyzed included hemoglobin (Hb), packed cell volume (PCV), and total erythrocyte count (TEC) [14].

### Statistical analysis

Adult worm mortality, body weight, EPG, and hematology data were analyzed using a one-way analysis of variance (ANOVA). The SEM and SDS-PAGE results were analyzed descriptively.

## Results

### Determining qualitative plant phytochemical

The quantitative phytochemical analysis showed that the leaves of *I. tinctoria* L. contained a number of secondary metabolites such as total tannins, saponins, flavonoids, and alkaloids (Table-1). The largest secondary metabolite compound was total tannins (5.43% w/w) and flavonoids (3.45% w/w).

**Table-1:** Quantitative phytochemical analysis.

Secondary metabolite compounds	%w/w
Total Tannins	5.43
Total Saponins	1.22
Total Flavonoids	3.45
Total Alkaloids	0.11

### *In vitro* study

Table-2 shows that IAE had a statistically significant effect on the mortality rate of *H. contortus* after 8 h of treatment (p<0.05). *H. contortus* deaths occurred after 8 h of immersion at a dose of 120 mg/mL and after 6 h at a dose of 160 mg/mL. In the negative control group, there was no worm mortality within 8 h of immersion. The most effective concentration that terminated adult *H. contortus* worms was obtained at a 220 mg/mL concentration, which resulted in 93.33% mortality (Table-2).

**Table-2:** Effect of IAE concentrations on the mortality rate of *Haemonchus contortus* after 8 h of immersion.

Treatment	Time of death (h)			

	2	4	6	8
NaCl 0.62%	0.00+0.00	0.00+0.00	0.00+0.00^a^	0.00+0.00^a^
IAE 100 mg/mL	0.00+0.00	0.00+0.00	0.00+0.00^a^	0.00+0.00^a^
IAE 120 mg/mL	0.00+0.00	0.00+0.00	0.00+0.00^a^	13.33+11.54^b^
IAE 140 mg/mL	0.00+0.00	0.00+0.00	0.00+0.00^a^	16.67+5.77^b^
IAE 160 mg/mL	0.00+0.00	0.00+0.00	10.00+17.32^b^	36.66+5.77^c^
IAE 180 mg/mL	0.00+0.00	0.00+0.00	16.66+20.81^b^	56.66+15.27^d^
IAE 200 mg/mL	0.00+0.00	0.00+0.00	33.33+28.8^c^	76.66+5.77^e^
IAE 220 mg/mL	0.00+0.00	0.00+0.00	56.66+15.27^c^	93.33+11.54^f^
Albendazole 1 mg/mL	3.33+5.77	96.66+5.77	100.00+0.00^d^	100.00+0.00^f^

^a,b,c,d,e,f^Different superscripts within the same column indicates significant differences (p<0.05). IAE=*Indigofera tinctoria* crude aqueous extract

**Figure-1:**
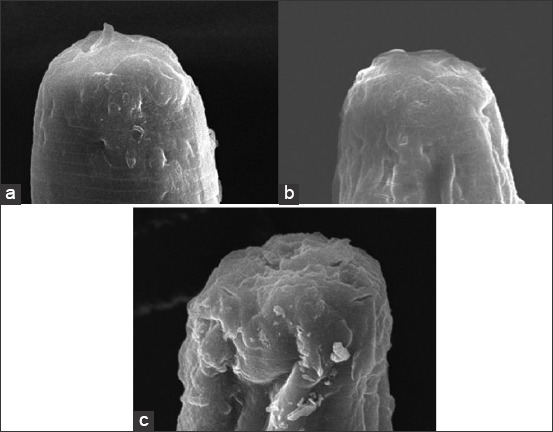
Ultrastructure of the anterior part of the worm: (a) Negative control. (b) A dose of 100 mg/mL: Anterior end was constricted. (c) A dose 220 mg/mL: Anterior end was constricted with thick folds.

**Figure-2:**
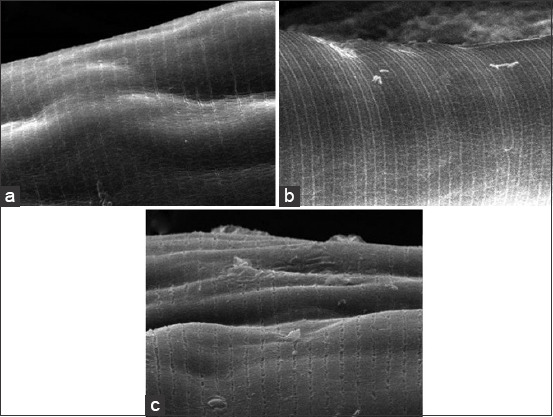
Ultrastructure of the midsection cuticle of the worm’s body: (a) Negative control. (b) A dose of 100 mg mL: Cuticle surface abraded. (c) A dose 220 mg/mL: The cuticle surface was wavy and abraded.

Figures-1 and 2 show the significant ultrastructural changes in adult *H. contortus* worms after *in vitro* immersion in IAE. In the control worms, their anterior part did not shrink, their cuticle had no aggregate, and there was no damage to the cuticle surface. However, damage occurred in the treatment worms (Figures-1, 2b, and 1, 2c). Their anterior end was wrinkled with thick folds, and their cuticles were abraded.

**Figure-3:**
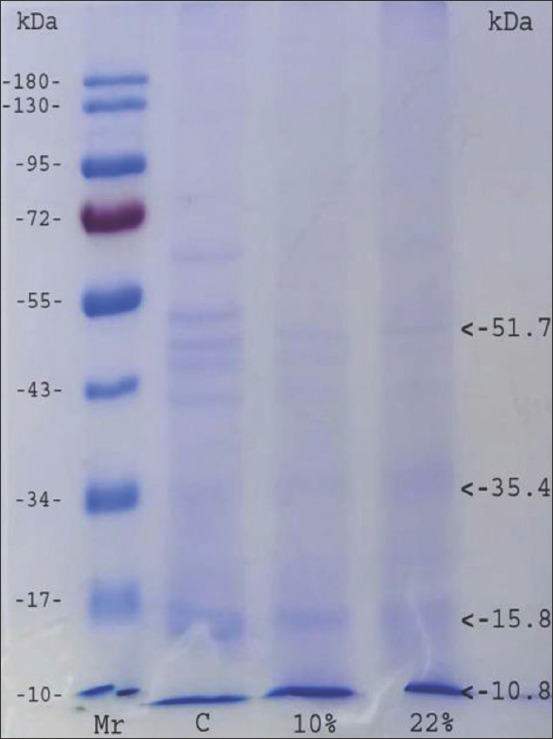
Sodium dodecyl sulfate-polyacrylamide gel electrophoresis electropherogram of *Haemonchus contortus*. Line 1 protein marker (Mr), line 2 worm protein of negative control (C), line 3, and 4 worm protein after immersion in 10% and 22% *Indigofera tinctoria* L. aqueous extract.

Figure-3 shows that the electrophoresis of the soluble protein of *H. contortus* after treatment produced five protein bands with molecular weights of 10.8, 15.8, 35.4, and 51.7 kDa in 22% IAE. Conversely, at a dose of 10%, there were six protein bands. There were eight protein bands with molecular weights of 10.8, 15.8, 35.4, 40.8, 47.0, 51.7, 56.9, and 72.1 kDa in the control group.

### *In vivo* study

**Figure-4:**
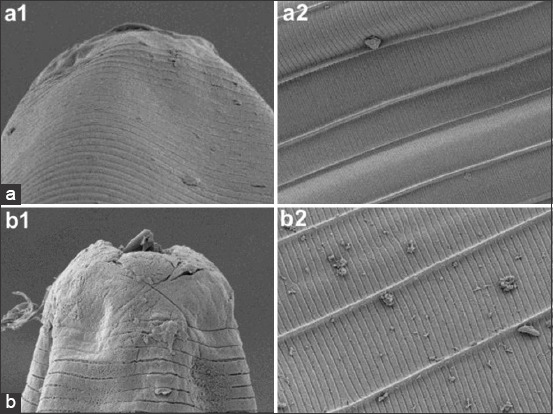
Ultrastructure of the anterior and the midsection part of the worm: (a) Negative control. (b) a dose 21 mg/mL.

Table-3 shows the highest FECRT value in the treatment group on the 14^th^ day after treatment at a dose of 62 mg/mL. The results of the ANOVA showed significant differences (p<0.05). Table-4 shows that the highest weight gain on the 14^th^ day after treatment at a dose of 41 mg/mL, which is 0.57 kg or 2.55%, and in the negative control, a weight loss of 0.40 kg or 2.08%. The highest increased Hb, PCV, and TEC values at day 14 were at a dose of 41 mg/mL, although the ANOVA did not show a significant difference (p<0.05; Table-5). The SEM results showed that IAE *in vivo* treatment caused the anterior part to shrink and aggregate to appear in the worm cuticle (Figure-4).

**Table-3:** The effect of IAE on fecal egg counts.

Dose of IAE (mg/mL)	Eggs per gram			Fecal egg-count reduction test (%)

	Pre-treatment	Post-treatment day 7	Post-treatment day 14	
Saline water 0.62%	1250.00±317.54^a^	666.67±44.10^a^	383.33±83.33^a^	69.36
21	800.00±200.00^a^	416.67±116.67^a^	50.00±50.00^b^	93.75
41	850.00±202.07^a^	583.33±216.67^a^	50.00±28.87^a^	94.12
62	750.00±325.32^a^	350.00±76.38^a^	16.67±16.67^c^	97.87
Albendazole	1383.33±231.54^a^	0.00±0.00b	0.00±0.00^d^	100

^a,b,c,d^Different superscripts indicate significant differences (p<0.05). IAE=*Indigofera tinctoria* crude aqueous extract

**Table-4:** The effect of IAE on sheep body weight (kg).

Dose of IAE (mg/mL)	Pre-treatment	Post-treatment day 79	Post-treatment day 14
Saline water 0.62%	19.25±0.70^a^	20.10±0.89^a^	18.85±0.60^a^
21	22.10±0.40^a^	22.42±0.52^a^	21.88±0.06^b^
41	22.33±0.68^a^	23.20±0.73^a^	22.90±0.89^b^
62	20.43±0.96^a^	20.80±1.00^a^	20.67±0.71^b^
Albendazole	21.55±1.68^a^	22.08±1.86^a^	22.08±1.52^b^

^a,b^Different superscripts indicate significant differences (p<0.05). IAE=*Indigofera tinctoria* crude aqueous extract

**Table-5:** The effect of IAE on sheep hematology.

Blood parameters	Dose of IAE (mg/mL)	Pre-treatment	Post-treatment day 7	Post-treatment day 14
Hb (g/dl)	Saline water 0.62%	12.56±0.73	12.40±0.30	12.47±0.64
	21	11.23±0.98	10.73±0.54	11.33±0.95
	41	9.83±0.82	11.23±0.39	12.43±0.34
	62	10.70±0.69	10.73±0.70	11.13±0.75
	Albendazole	10.30±0.35	9.90±0.0.11	11.00±0.30
PCV (%)	Saline water 0.62%	35.10±1.95	36.50±1.31	35.47±1.74
	21	32.63±3.28	31.20±1.70	33.00±2.56
	41	29.67±1.51	33.57±0.99	35.97±1.81
	62	32.73±2.44	32.50±2.51	32.23±1.98
	Albendazole	31.57±1.14	29.93±1.23	32.47±1.46
TEC (million/UI)	Saline water 0.62%	12.92±0.84	13.45±0.50	13.24±0.88
	21	12.12±0.67	11.83±0.63	12.32±0.59
	41	10.04±1.97	11.34±1.41	12.15±0.89
	62	10.99±0.15	10.88±0.64	11.00±0.92
	Albendazole	10.55±1.15	10.12±0.69	11.04±0.73

Overall values do not differ significantly (p<0.05); Hb=Hemoglobin, PCV=Packed cell volume, TEC=Total erythrocyte count, SE=Standard error

## Discussion

The quantitative phytochemical analysis showed that *I. tinctoria* L. contained secondary metabolites with the highest amount of total tannins and flavonoids. The previous studies conducted by Rahman *et al*. [16] and Verma and Suresh [17] found that *I. tinctoria* leaves contain flavonoids and alkaloids. Mishra *et al*. [18] also found that tannins and saponins are present in *I. tinctoria*. Tannins, saponins, flavonoids, and alkaloids in the extract of *P*. *falcaria* have previously been shown to have an anthelmintic effect against *H. contortus* [19]. Klongsiriwet *et al*. [20] added that the combination of flavonoids and tannins had a synergistic anthelmintic effect against *H. contortus*. Joshua *et al*. [11] stated that the medicinal potential of plant leaf extracts could be identified from their phytochemical components.

In the current study, the highest mortality in *H. contortus* occurred after 8 h of immersion, which was believed to be caused by the reaction of the collective presence of bioactive compounds in the plant extracts. This study’s findings are similar to those of Ferreira *et al*. [21], who reported that the leaf extract of *Annona muricata* L. could completely inhibit the motility of adult *H. contortus* worms in the first 6-8 h. Ahmed *et al*. [22] stated that *Artemisia herba-alba* and *Punica granatum* had a significant effect on the mortality rate of adult *H. contortus* after 7 h of exposure. Zaman *et al*. [23] reported that herbal complex extracts could cause 100% death in adult *H. contortus* worms 8 h post-exposure. Widiarso *et al*. [24] stated that the infusion of *Gigantochloa apus* could cause 100% mortality against *H. contortus* after 4 h of immersion. *H. contortus* adult worms’ mortality is an effect of the tannins in the plant extract. Tannins can directly affect adult *H. contortus* worms by impairing the protective function and absorption of *H. contortus* [25]. Maestrini *et al*. [8] added that saponins could cause death by increasing the cell membrane permeability. Alkaloids work by affecting the activity of the worm muscles [26].

The damage to the anterior end and cuticle that experienced abrasion proved that IAE interacted with the nematode cuticle. Baihaqi *et al*. [19] stated that an *in vitro* extract of *P. falcataria*’s bark waste caused damage to the buccal area, and aggregate built up in the cuticle annular. Acevado-Ramírez *et al*. [27] stated that *Castanea sativa* caused damage around the mouth, anus, vulva, copular bursa, and cuticle of worms. Martínez-ortíz-de-montellano *et al*. [28] added that the leaf extracts of *Onobrychis viciifolia* and *Lysiloma latisiliquum* caused changes in the transferal fold and thickening of the cuticle of adult *H. contortus* worms. Yoshihara *et al*. [29] added that *Acacia mearnsii* extract caused ruptures of the cuticle and transverse wrinkles on the body surface of adult *H. contortus*. Barone *et al*. [30] added that *Cranberry vine* extract caused aggregate accumulation in the cuticle and buccal areas of *H. contortus*. The peeling and wrinkling of adult *H. contortus cuticle* after soaking with *Biophytum petersianum* were reported by Sambodo *et al*. [10]. Tresia *et al*. [31] stated that cuticle damage and changes in the permeability of the worm cuticle were related to the active ingredients found in plants. Damage to the cuticle disrupts the protective function and absorption of nutrients, resulting in malnutrition and impaired motility, which causes the worms to die. The mechanism of cuticle damage occurs through the insertion of tannins into the lipid layer in the cuticle (glycosylation) and interaction with the proline and hydroxyproline collagen layers in the cuticle, resulting in changes in the cells [32].

Soaking *H. contortus* in *I. tinctoria* extract showed a decrease in the number of protein bands. Several studies have shown different numbers of protein bands. Sambodo *et al*. [10] stated that *H. contortus* soaked in a 10% dose of *B. petersianum* showed five protein bands with sizes of 9.3, 17.1, 50, 63.2, and 72.7 kDa. Mubarokah *et al*. [15] stated that *A. catechu* extract decreased the number of protein bands. Jaiswal *et al*. [33] added that in *H. contortus* females, there were 35 protein bands. In this study, the decrease in the number of protein bands was caused by secondary metabolites that disrupted the protein binding in the worms. Hoste *et al*. [34] stated that tannins could bind proline and hydroxyproline, while worm bodies are known to contain lots of proline and hydroxyproline. Zong *et al*. [25] added that tannins binding to proteins affected the physiological processes of worms and/or modified the host immune response in eliminating adult worms.

The decreased EPG value after treatment was the same as the results of research conducted by Simon *et al*. [35], who stated that *Combretum molle* extract decreased the FEC in sheep infected with *H. contortus*. González-Cortazar *et al*. [36] stated that the aqueous extract of *L. acapulcensis* leaves reduced the EPG value of gastrointestinal nematodes in sheep. Sambodo *et al*. [14] also stated that the aqueous extract of *Biophytum petersianum* decreased the EPG value of *H. contortus* in goats. The decrease in the EPG value is believed to be caused by secondary compounds in *I. tinctoria*. The presence of flavonoids, tannins, saponins, and alkaloids in *C. molle* may be responsible for the anthelmintic effect of these plants [35]. González-Cortazar *et al*. [36] added that myricitrin (a flavonoid) is the main component for reducing the EPG value.

The treatment group’s increased body weight is believed to be due to the loss of most of the worms in the abomasum of the treated animals so that the absorption of nutrients from the feed was improved. These results are consistent with those of Sambodo *et al*. [14], who stated that *B. petersianum* extract increased the body weight of goats infected with *H. contortus*. Eguale *et al*. [37] stated that this could occur in livestock due to reduced blood plasma and protein associated with the presence of parasites.

The blood test results in this study were similar to those of Meenakshisundaram *et al*. [38], who stated that the ethanol extract treatment of *I. tinctoria* increased the Hb, PCV, and TEC values in sheep infected with gastrointestinal parasites. Eguale *et al*. [37] stated that *Coriandrum sativum* extract increased the PCV value 7 days after treatment. The increased hematological values are believed to be due to the loss of worm parasites in the abomasum so that the blood components can be better absorbed by the body. The increased Hb is probably due to increased iron absorption [38].

The SEM results at an *in vivo* stage are consistent with the SEM results at the *in vitro* stage. Changes in the ultrastructure of helminth parasites prove a strong interaction with IAE. This study’s results are similar to those of Martínez-ortíz-de-montellano *et al*. [28], which found that *Tzalam* leaf extract causes aggregates in the anterior end of *H. contortus*.

## Conclusion

IAE contains tannins, saponins, flavonoids, and alkaloids. Aqueous extract of *I. tinctoria* L. has an anthelmintic effect, resulting in decreased FEC, increased weight gain, Hb, PCV, and TEC, causing damage to the worms’ body and reducing the protein profile of adult *H. contortus* worms.

## Authors’ Contributions

JP and IM: Designed and managed the study. IM: Collected and analyzed the samples. IM and SS: Wrote the manuscript. JK and WN: Reviewed the manuscript. All authors have read and approved the final manuscript.
